# Supplementary material for: Structural Eigenmodes of the Brain to Improve the Source Localization of EEG: Application to Epileptiform Activity
Source: Adv Sci (Weinh). 2026 Mar 23;13(31):e16802. doi: 10.1002/advs.202516802 (PMC13588046; doi:10.1002/advs.202516802)
Supplement: Supplementary file 1 [file ADVS-13-e16802-s001.pdf]

Supporting Information

Structural Eigenmodes of the Brain to Improve the Source Localisation of EEG: Application to Epileptiform Activity

DOI: 10.1002/advs.202516802

Pok Him Siu[1,2,*], Philippa J. Karoly[1,2], Sina Mansour L.[1,3], Artemio Soto-Breceda[4], Levin Kuhlmann[5], Mark J. Cook[1,2,6], David B. Grayden[1,2,6]

[1] Department of Biomedical Engineering, The University of Melbourne, Australia
[2] Graeme Clark Institute, The University of Melbourne, Australia
[3] National University of Singapore, Singapore
[4] Grenoble Institut des Neurosciences, INSERM, France
[5] Monash University, Australia
[6] Department of Medicine, St Vincent's Hospital, Melbourne, The University of Melbourne, Australia

[*] Corresponding author: pokhim.siu@student.unimelb.edu.au

S1 Supplementary Material

S1.1 Simple Source Simulations

For a more direct comparison with previous literature which focuses typically on one to few sources of various extent [21, 24, 25, 26, 27, 28], we also had a simple set of single-source simulations. These sources were generated with varying sizes such that for each simulation, one cortical region of the 1000-region parcellation was selected as the centre. This simulation would also be more similar to the case of averaged, interictal spikes. We compared the performance of geometric eigenmodes without weighting, with weighting, and against eLORETA. The geometric eigenmode approach had superior performance to eLORETA at the single-patch source level. The significant improvement in metrics with weighting mirror previous results stating that low-wavelength activity dominate cortical activity [9].

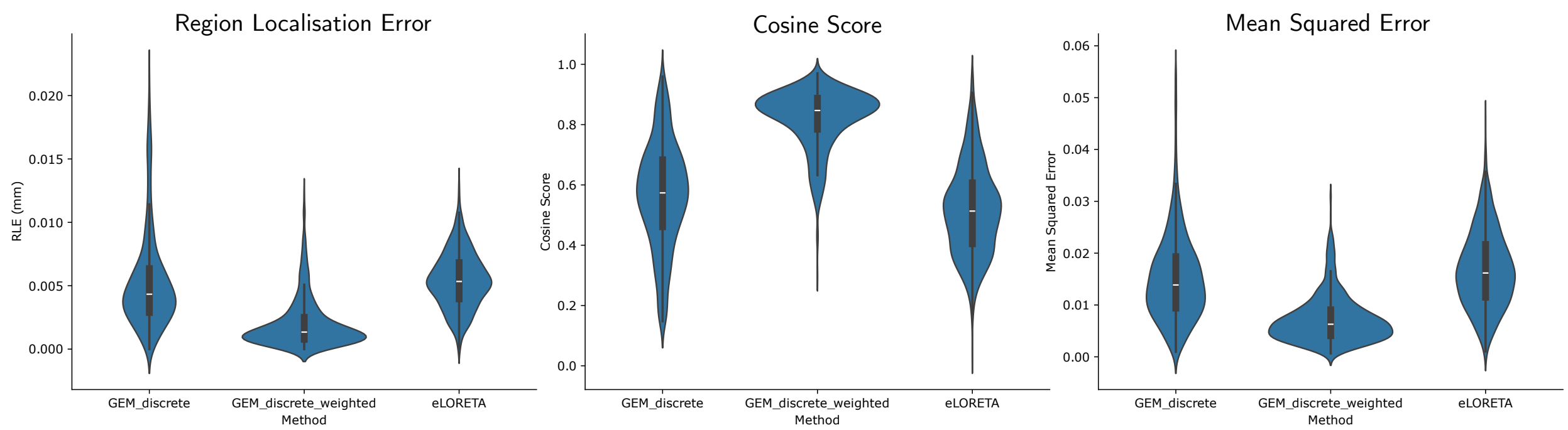

Figure S1: Patch simulations demonstrating performance of: i) GEMs with no weighting, ii) GEMS with eigenvalue weighting, iii) eLORETA. Sources had fixed orientations.

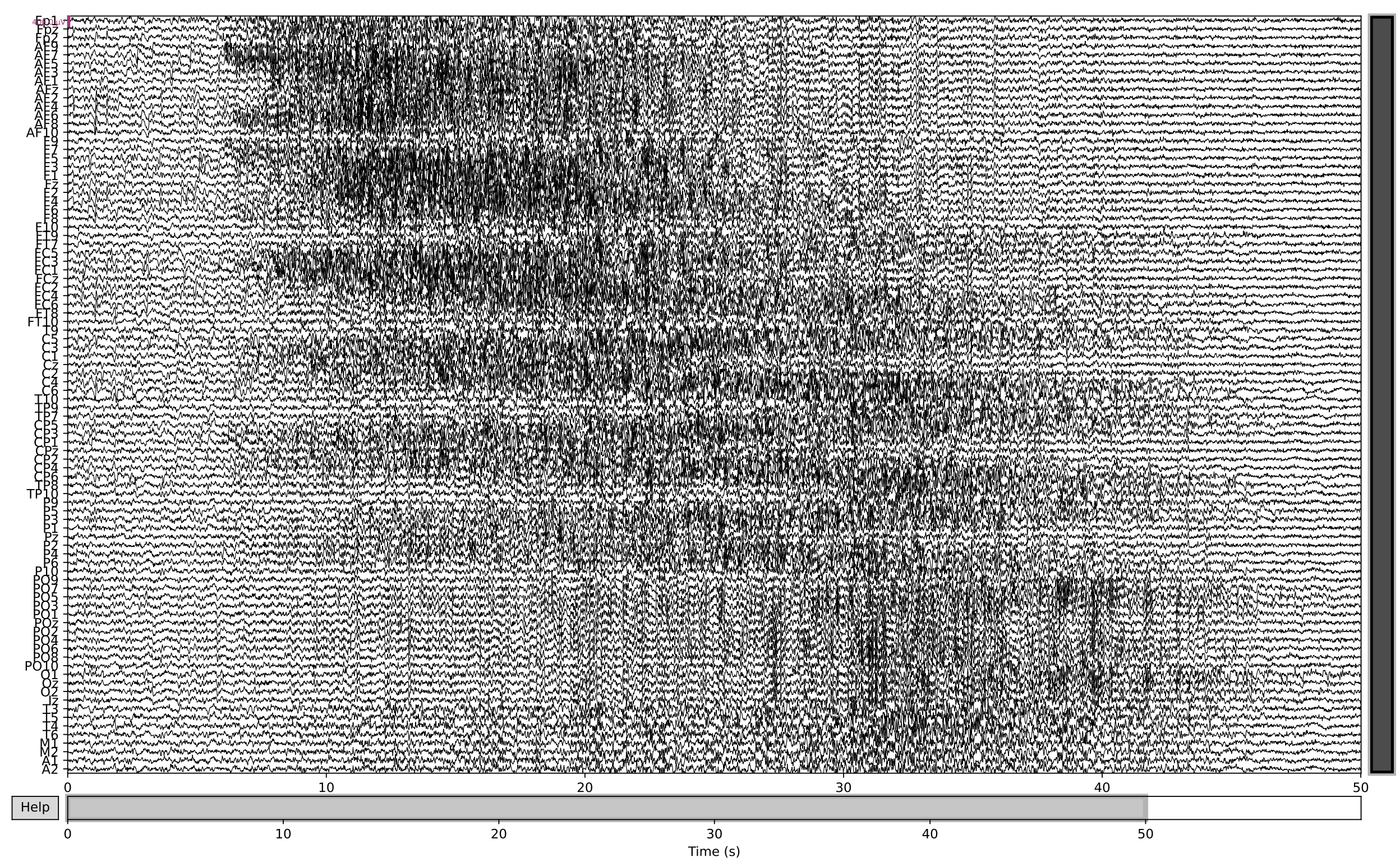

Figure S2: EEG trace of simulation with SNR of 10 dB. The simulated EEG exhibited clinically consistent features, including focal onset, spatial spread, and temporal evolution with characteristic slowing.

S1.2 Connectome Eigenmode Weighting and Combined Structural Eigenmodes

Per Eq. 8, geometric eigenmodes were weighted by $\beta = 1/2$ and connectome eigenmodes were weighted by $\beta = 1$ so that the weights ended up being very similar after normalisation as demonstrated in Fig.S3. The regularisation constant was selected to be the same for both to ensure that the overall weights were similar. These are the weights used when testing geometric and connectome eigenmodes together as the basis set for source localisation, which does not appear to offer a substantial difference (see Fig.S4). Explicitly, Fig S3 is graphing:

$$W_G = \frac{\pi}{0.1 + |\frac{\lambda_G}{\bar{\lambda}_G}|^{1/2}} \tag{18}$$

$$W_C = \frac{\pi}{0.1 + |\frac{\lambda_C}{\bar{\lambda}_C}|} \tag{19}$$

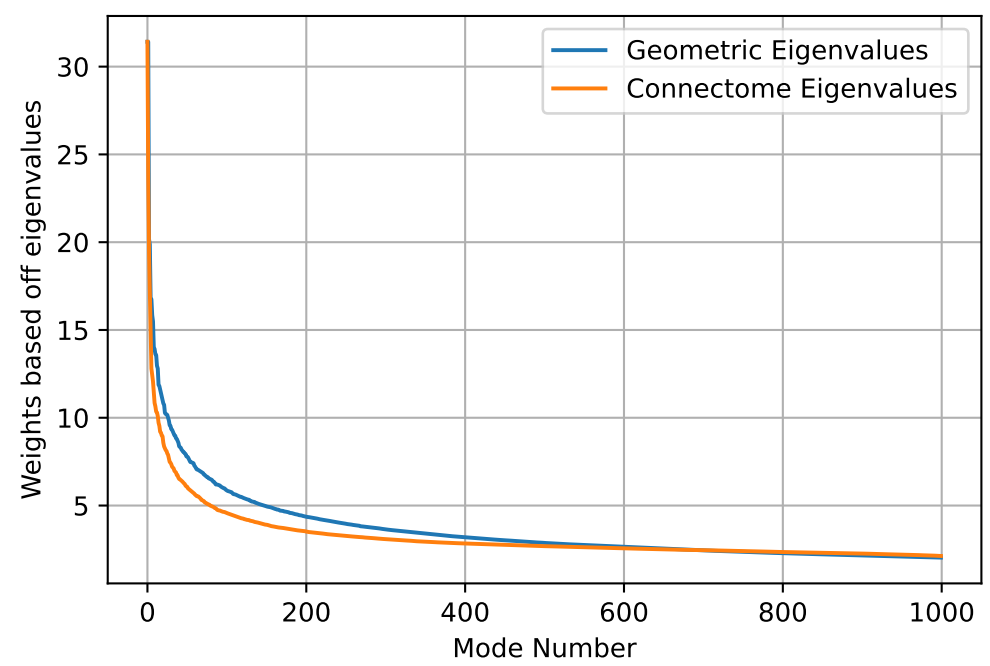

Figure S3: Visualising the weights after eigenvalue weighting. Noting that a different power for the eigenvalue was applied so that the GEMs and CEMs have similar weights.

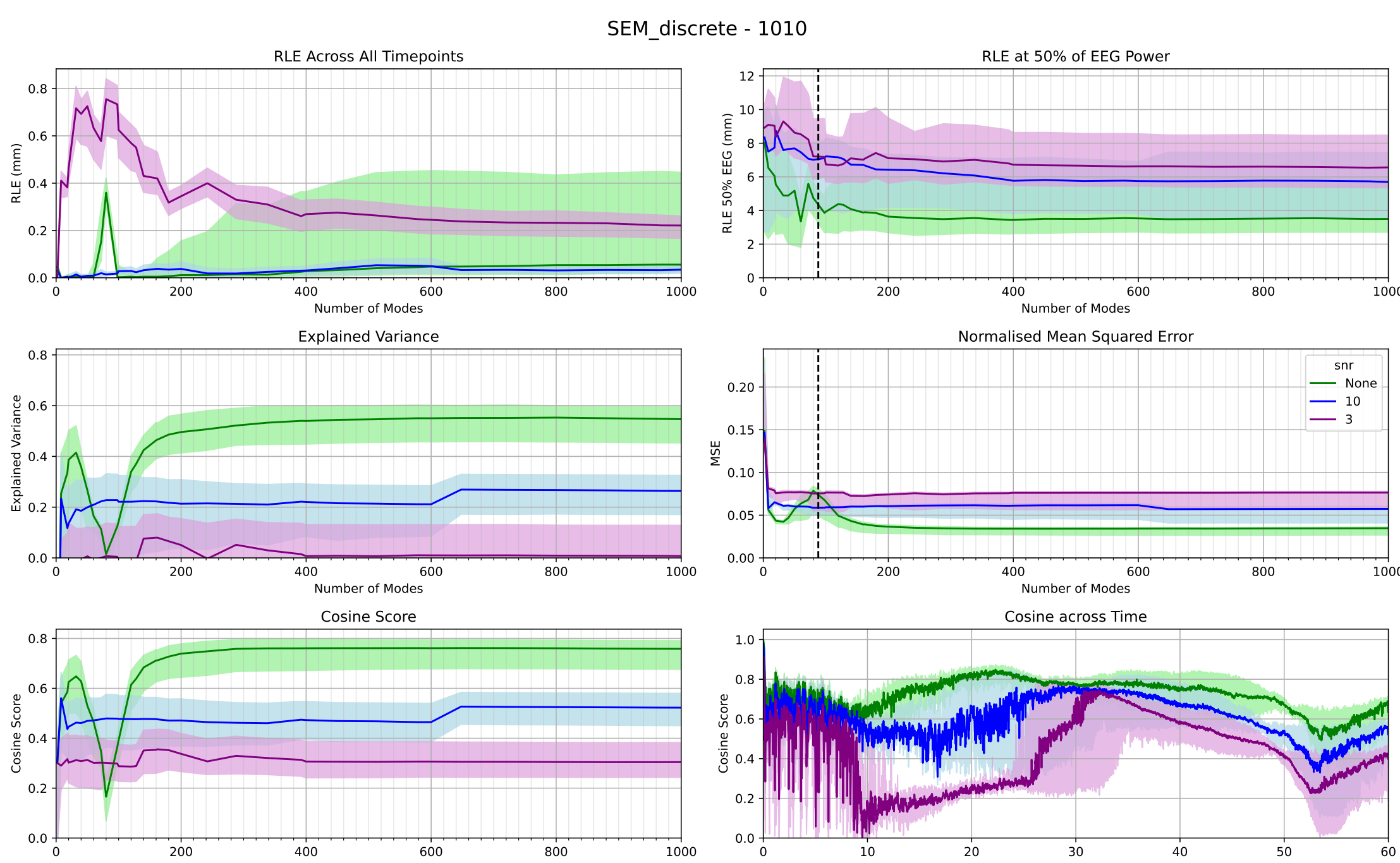

Figure S4: Performance for the structural eigenmode approach when concatenating the set of modes of geometric and connectome eigenmodes after weighting as per Fig.S3.

S1.3 High-density Source Space

Within the main text, the analysis focused on a 1000-vertices source space which matched the spatial resolution of the underlying discrete connectome. Here we present analysis of the structural eigenmode source localisation approach performed on icosahedron-5 (ico-5) source spacing consisting of 20 484 dipole sources. In ico-5 space, multiple dipoles are associated with each cortical region and the underlying source time series are projected from 1000 cortical regions to the 20 484 sources. Furthermore, Laplacian smoothing of neighbouring values is applied with 5 iterations to remove boundary effects of the projection. The corresponding EEG signal is then calculated with a forward transform from the ico-5 space. To perform high-resolution connectome eigenmode analysis, the eigenmodes were constructed on a high resolution connectome in fsLR space and subsequently mapped to ico-5 source spacing [41, 83].

Both the geometric eigenmodes and the connectome eigenmodes performed reasonably well in this high-density source space, with similar trends found in the 1000-vertices source space. At this high-density source space, a more notable distinction between geometric eigenmodes and its rotational surrogates emerges, and the connectome eigenmodes perform better than the geometric eigenmodes' rotational surrogates. See Fig. S5 for the comparison of metrics, and Fig. S6 for a visualisation of the source estimates.

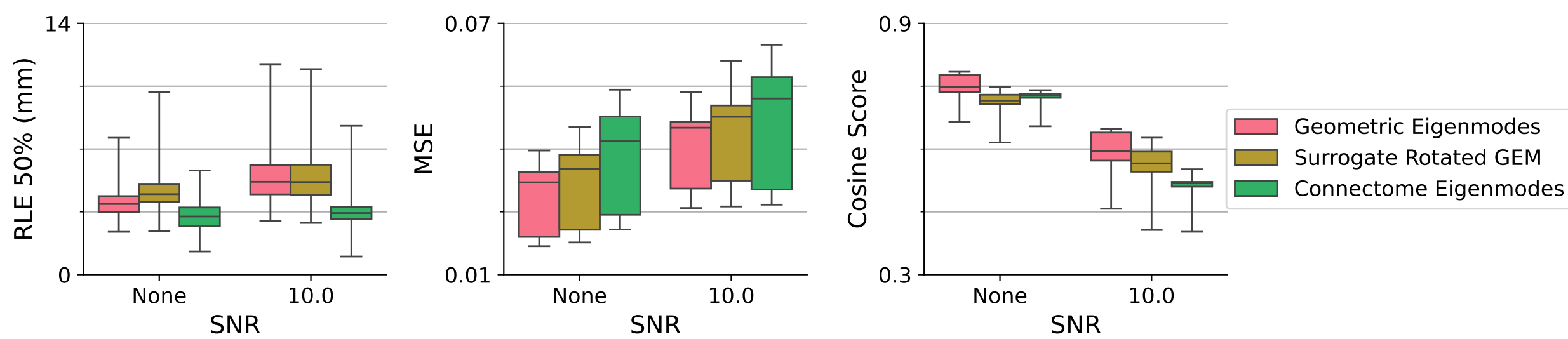

Figure S5: Structural eigenmode approach performed on ico-5 source space consisting of 20 484 sources. In ico-5 space, multiple dipoles are associated with each cortical region and the underlying source time series are projected from 1000 cortical regions to the 20,484 sources. The corresponding EEG signal is then calculated with a forward transform from the ico-5 space. Both GEMs and CEMs performed reasonably well in this high density source space.

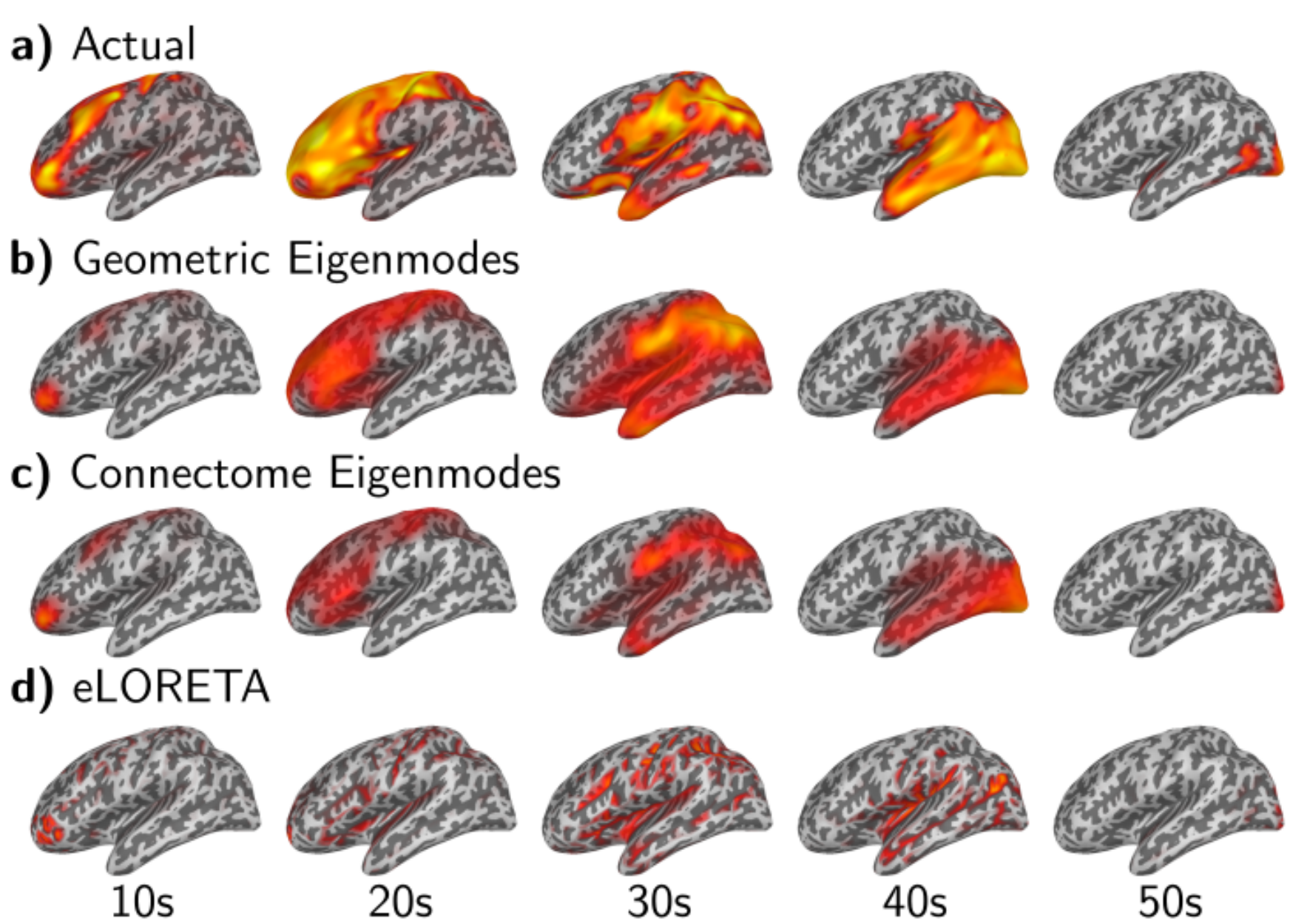

Figure S6: Source localisation example for seizure originating from thalamus plotted on left hemisphere in ico-5 source space consisting of 20 484 sources.

S1.4 Tuning the Truncation Parameter at High Noise Levels

The reader may observe in Fig. 6 that the performance of GEMs decreases more than the performance of CEMs when the SNR is low (at 3 dB). We split our response to this observation into two parts: i) an explanation of the mechanisms behind the discrepancy, ii) the practical solution to overcome the issue.

The reason for the observation is a combination of two factors: i) regularisation/truncation for noise, and ii) differences in the spatial scales of CEMs and GEMs. As detailed in the last paragraph of Section 2.3, in this work, we use SVD (singular value decomposition) to solve the underdetermined inverse problem. A reciprocal conditioning number (rcond parameter) is used to truncate modes with small singular values, thereby reducing noise amplification. With increasing noise, more modes are truncated. As a result of using a heuristic for this parameter, a substantial number of modes are truncated at low SNR, and the number of retained components is similar between CEMs and GEMs.

The difference then lies in the difference in spatial scales between early modes of CEMs and GEMs, depicted by the eigenvalue spectra. At early modes, connectome eigenmodes encompass a broader range of spatial wavelengths than geometric eigenmodes. Under conditions of heavy truncation, this difference in spatial scale becomes consequential. When only a small number of components are retained, CEMs preferentially preserve broader spatial structure, whereas GEMs are more affected by the loss of intermediate-scale structure. This interaction between truncation and spatial scale helps explain why CEMs seemingly exhibit greater robustness under high-noise conditions (strict regularisation) in our experiments.

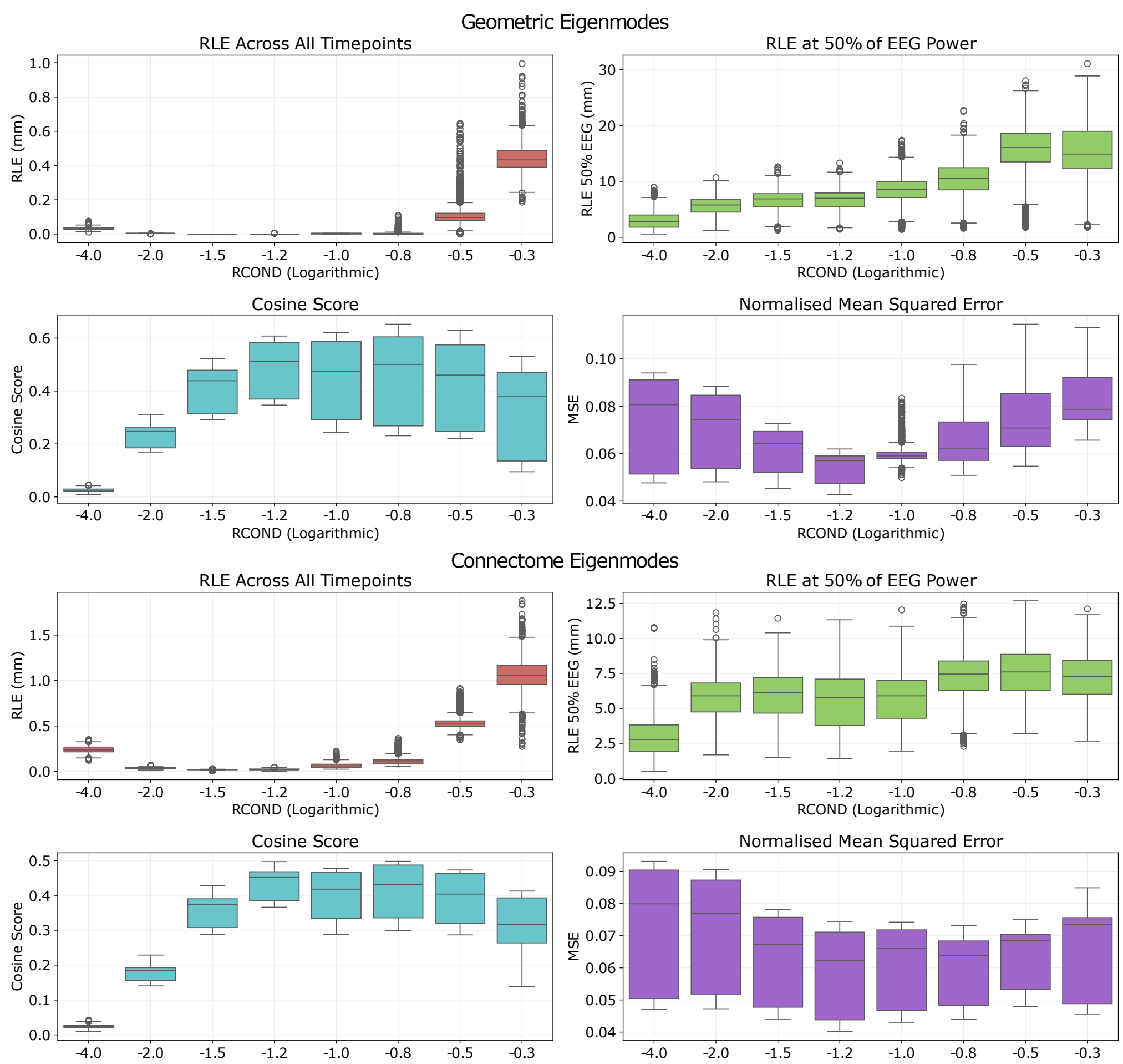

Figure S7: Geometric Eigenmode and Connectome Eigenmode Results at SNR 3 Under Different Truncation Values. The x-axis has logarithmic values. Given our regularisation formula was $\mathrm{rcond} = 10^{(\mathrm{SNR}/10)}$, the "equivalent" SNR truncation level would be the rcond value multiplied by –10.

The solution would be for a more sophisticated truncation schema, for example, by setting a lower bound on the number of modes truncated (an upper bound for rcond). Figs. S7 graph the source imaging results across different rcond values for both GEMs and CEMs at SNR = 3. Per our heuristic, rcond was set to $10^{-0.3}$, which ended up truncating a few too many modes. From the two figures above, a more ideal minima would have been between $10^{-1.2}$ and $10^{-0.8}$ for our specific dataset for both GEMs and CEMs. A rcond of $10^{-1.2}$ would align well with the first peak in performance when varying the number of modes in Fig. 5. Notably, GEMs appear to still have slightly better performance than CEMs here, just as in previous results for higher SNR.

Tuning the regularisation parameter is done in practice, such as in similar related work [20], but we believe that for a fair comparison across different methods and to avoid overfitting, it was important to use a heuristic. Furthermore, in a real-world situation, we would not have had the underlying source of truth. We did not wish to unintentionally bias one method to be better than another.

S1.5 Optimised Comparison with Prior Eigenmode-Based Source Inversion Methods

To further contextualise the proposed approach, we performed an additional comparative analysis against previously proposed eigenmode-based source inversion methods, specifically those of Wang et al. [19] and Rué-Queralt et al. [20]. In the main manuscript, we chose to compare against established baseline methods from the LORETA family, as these approaches are widely used, well understood, and provide a reliable reference point for evaluating practical source localisation performance. It should be noted that methods from the LORETA family remain the most widely used approaches in practical EEG/MEG source imaging, particularly in clinical and translational settings. Nevertheless, comparison with recent eigenmode-based methods is informative for assessing performance within the same methodological family and this is presented as Fig. S8.

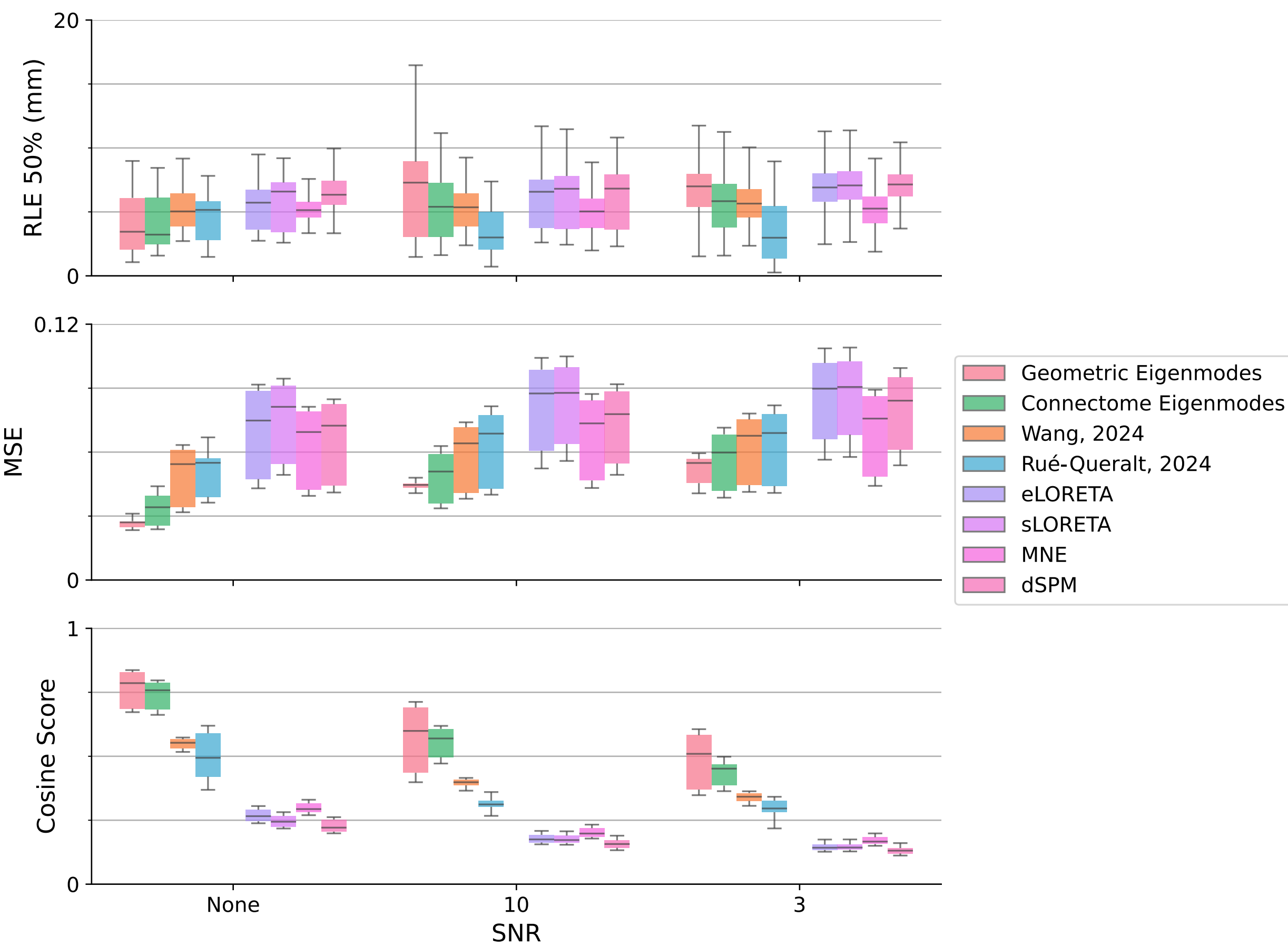

Figure S8: Comparison of structural eigenmode approaches in literature under optimised conditions

As neither Wang et al. [19] nor Rué-Queralt et al. [20] provide publicly available reference implementations (with the latter additionally reporting a patented methodology), the present comparison is based on our independent re-

implementation of the published descriptions. While every effort was made to faithfully reproduce the reported methods, we present these results in the Supplementary Material to avoid over-interpretation of results.

We note that the compared methods differ substantially in their optimisation frameworks. In particular, Rué-Queralt et al. [20] employ an L1-regularised formulation with grid-search optimisation of the regularisation parameter, which is computationally demanding and requires explicit tuning across signal-to-noise ratio levels. To ensure a fair comparison, we therefore applied automatic regularisation tuning to all methods in this supplementary analysis. As a result, performance across all methods is slightly improved relative to the heuristic-based regularisation used in Fig. 5 of the main manuscript.

The observed performance differences can be understood as arising from two primary methodological distinctions: i) the eigenvalue weighting strategy employed in our approach, and ii) the use of an SVD-based truncated pseudo-inverse framework, which allows weighting and truncation to be treated separately rather than being encapsulated within a single regularisation hyperparameter. This added flexibility appears to be beneficial under varying noise conditions.

S1.6 Different Number of EEG Electrodes

Within the main text, we focused our analysis on 10-10 spacing corresponding to 88 EEG electrodes as this is a common number used in research. We have also investigated 10-05 spacing corresponding to 343 electrodes (Fig. S9 and 10-20 spacing corresponding to 21 electrodes (Fig. S10).

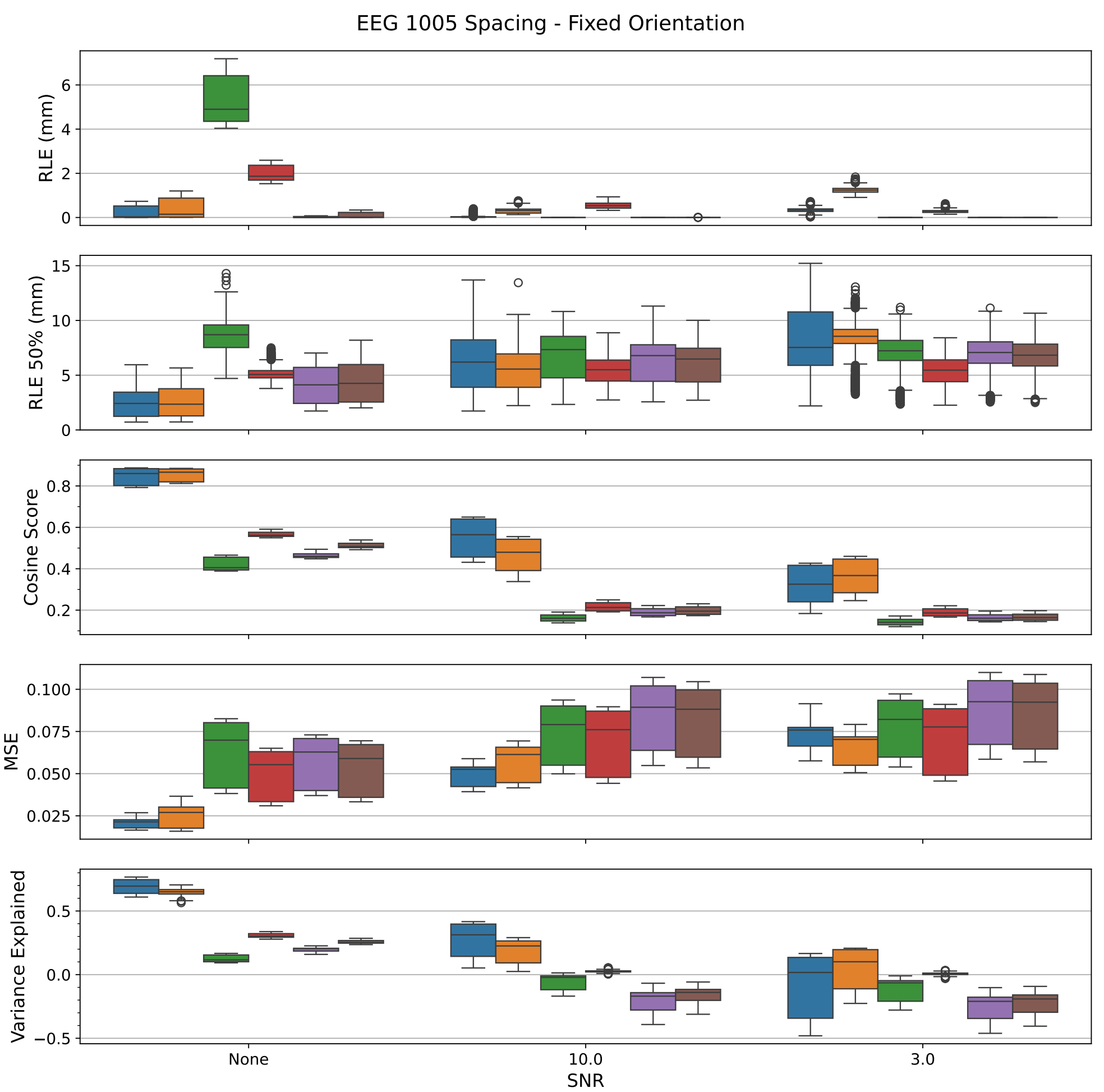

Figure S9: EEG 10-05 spacing metrics for fixed orientation. Legend: Blue = GEM, Orange = CEM, Green = dSPM, Red = MNE, Violet = sLORETA, Brown = eLORETA.

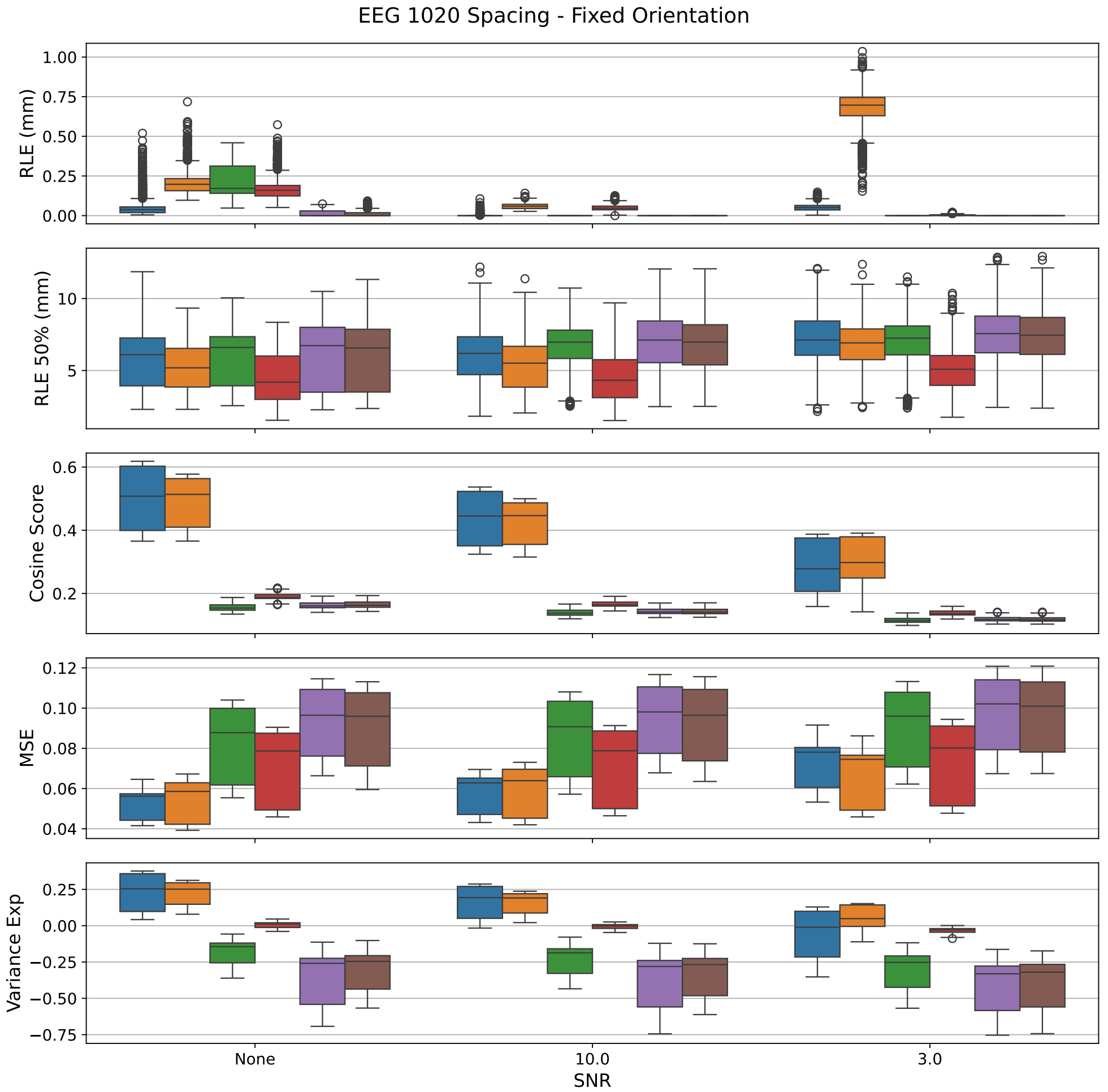

Figure S10: EEG 10-20 spacing metrics for fixed orientation.

Key highlights would be both geometric eigenmodes and connectome eigenmodes were able to reasonably resolve sources at all electrode configurations. The algorithms did not perform well at a SNR of 3. At 21 and 88 electrodes, the MN methods were unable to accurately determine the polarity of sources, as evidenced by the variance explained being close to 0, and the relatively low cosine score. At 343 electrodes, the MN methods were able to resolve the polarity of sources, leading to substantial increases in metrics.

S1.7 Allowing Loose Orientations of Dipoles

Allowing the orientation of the dipole to be free to deviate from the normal of the cortical surface (also referred to as loose orientation) is a common approach in source localisation [50, 84]. As previously stated, in this work the dipoles were oriented normal to the cortical surface for the simulation. However, preliminary results suggest that allowing a deviation from the normal of the cortical surface, by setting the 'loose' parameter to $! = 0$, increased performance for all source localisation methods, and the LORETA family more significantly. The standard explanation would be that the loose orientation provides extra degrees of freedom to account for small errors with the forward model, mesh discretisation, downsampling, etc. This section is to provide evidence that such a performance increase can be deceptive and that the performance increase is primarily a result of condensing the vector source estimate into a magnitude measurement, rather than any error correction.

First, let us demonstrate how setting a loose orientation appears to improve source localisation performance. This is as seen by comparing Fig.6 with Fig.S11. For the EM methods, this improved the handling of noise, but decreased performance slightly under no noise conditions. For the LORETA family, this significantly improved overall performance for all SNRs.

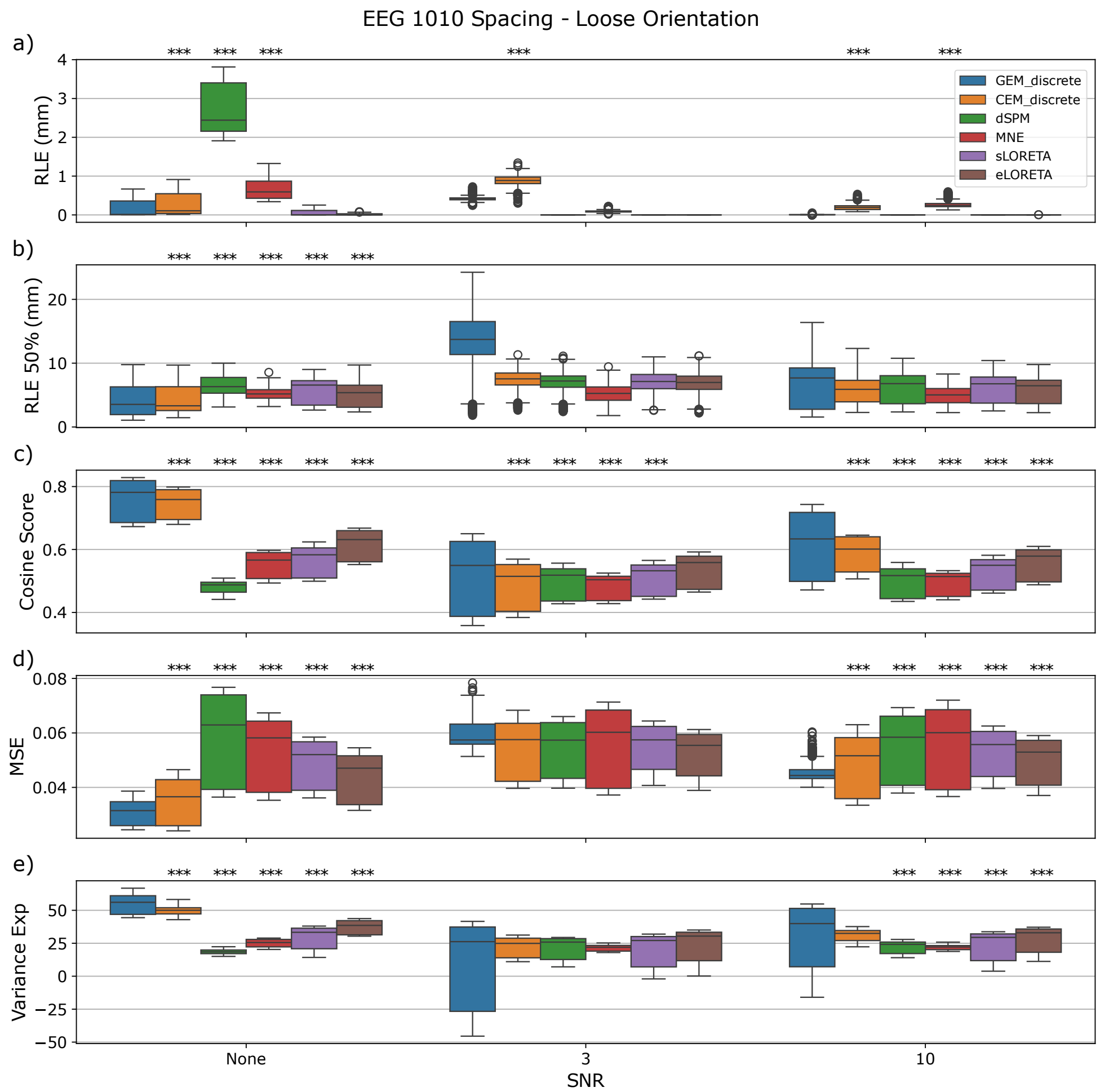

Figure S11: Metrics for loose orientation

However, the apparent improvement in performance can be demonstrated to be an artifact of vector estimation. After estimating the loose source estimate, there is a need to compare it with the true source estimate which is oriented normal to the cortical surface. The typical method, as default in MNE-Python, is to condense the vector into a magnitude and compare, and this is what was performed to generate Fig. S12c)-d) and Fig. S13c)-d). Both figures plot source estimates from a single simulation (seizure originating from Thalamus at $g = 2$). Sequentially, a) represents fixed orientation, b) represents fixed orientation but then taking the absolute value of source estimate, c) represents setting a loose orientation of 0.05, and d) represents loose orientation of 1. It can be seen that under these conditions, adding the loose parameter significantly increased performance. Further increase of the loose parameter appears to slightly increase the performance for eLORETA. Fig.S13b) demonstrates that the performance increase can also be seen by simply taking the absolute value of the fixed orientation estimate. Essentially, what this means is that the removal of the direction (and only taking the magnitude) is producing the bulk of the performance increase, rather than the allowance of deviation to the cortical surface. Indeed, Fig.S13e) displays the estimate and metric when only considering the orientation component normal to the surface results in relatively poor performance. Given that the true sources are known to be fixed-orientation, the misleading gains by allowing loose orientation is why in the main text we present results in the fixed orientation source space.

In EEG and MEG source localisation, polarity reflects the orientation of the underlying current dipole relative to the sensors. Although this orientation is faithfully preserved in the forward model, inverse solutions may fail to recover it because multiple source configurations can explain the same sensor pattern. As a result, many localisation pipelines prioritise spatial accuracy while treating polarity as a secondary or unstable feature [50]. For applications such as identifying the epileptogenic zone (or, more broadly, any task focused on where activity originates), spatial

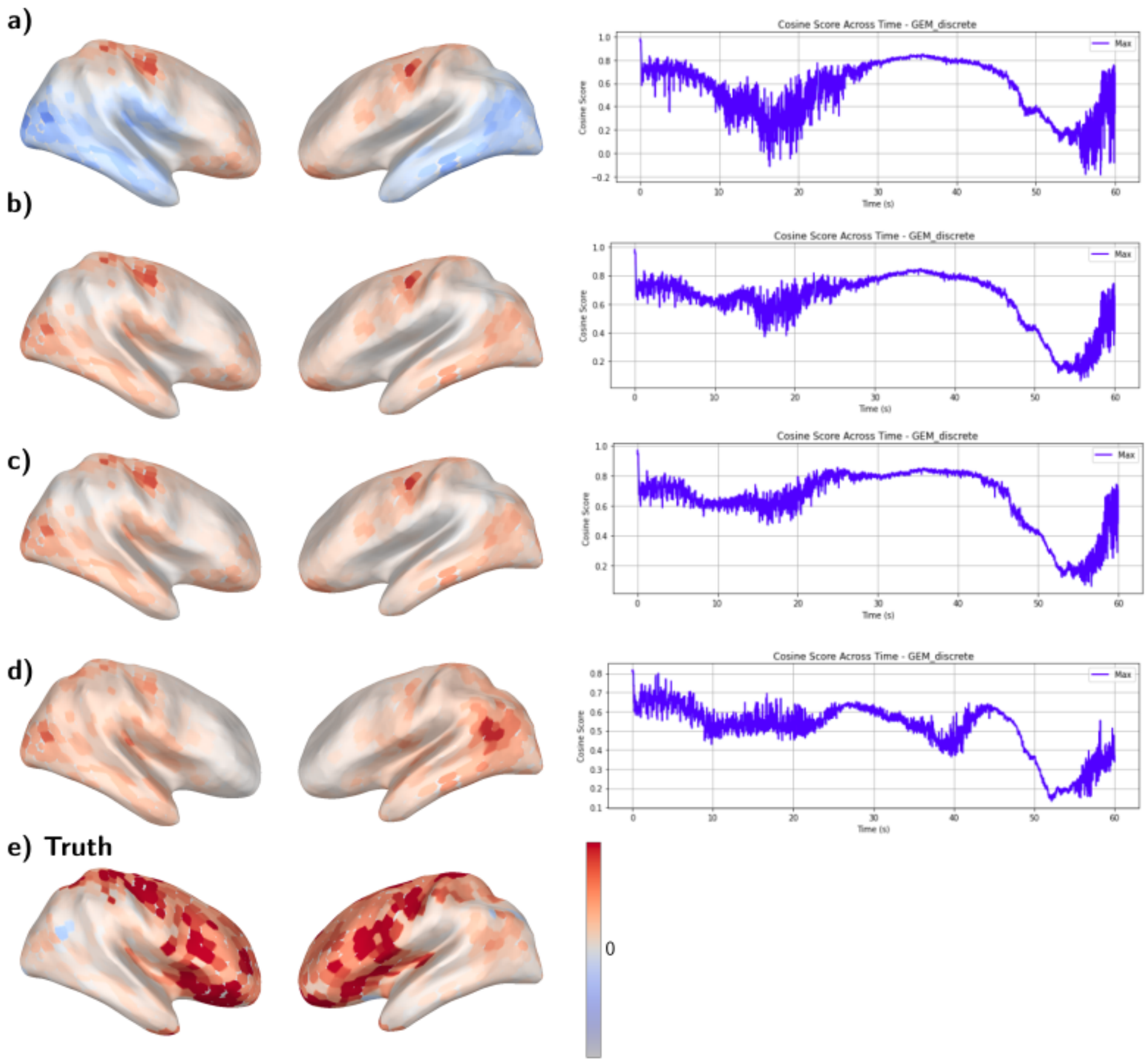

Figure S12: GEM method adjusting the loose parameter of a thalamic seizure with SNR 10. The localisation result at t=20s is visualised on the brain surface as it is a timepoint of unstable and relatively poor performance. The cosine score across the entire simulation is plotted beside. a) Loose = 0 (fixed orientation). b) Loose = 0, with np.abs(estimate). c) Loose = 0.05 (loose orientation). d) Loose = 1 (free orientation). e) True source, noting that the colourscale is diverging, whereas main text plots have been one-sided. Comparing a) with b), under noisy conditions, the algorithm may incorrectly estimate the correct polarity. Between 50s and 60s, a few sparse sources are active, and noise causes the algorithm to estimate the location incorrectly, noting that cosine score does not account for spatial similarity in that an estimate that is off by 1cm is treated the same as if it was off by 10cm.

precision is indeed far more critical than recovering dipole orientation, especially given susceptibility of polarity to noise, head-model inaccuracies, and orientation ambiguities, as demonstrated in the preceding simulations.

Nevertheless, polarity can carry meaningful physiological information. The polarity represents the direction of current flow across the cortical surface and through the cortical laminae, with implications on the hierarchical (such as feedforward or feedback) relationships between areas in the brain [85, 86, 87]. Polarity may also be essential when interpreting functional connectivity [88, 89], phase relationships [8, 33], or propagation direction [33]. Therefore, incorrect signs can invert inferred interactions or misrepresent the temporal ordering of neural activations. As such, the importance of resolving the correct polarity of the dipole sources should be guided by the scientific or clinical question being asked.

S1.8 The Difference between CEM-based and GEM-based Representations

Connectome eigenmodes are constructed from the connnectivity matrix, whereas geometric eigenmodes are constructed from a surface mesh. Clearly, the two types of eigenmodes differ in their construction and origins. However, in the main text, we found that both types of eigenmodes offered similar levels of performance when incorporated into a

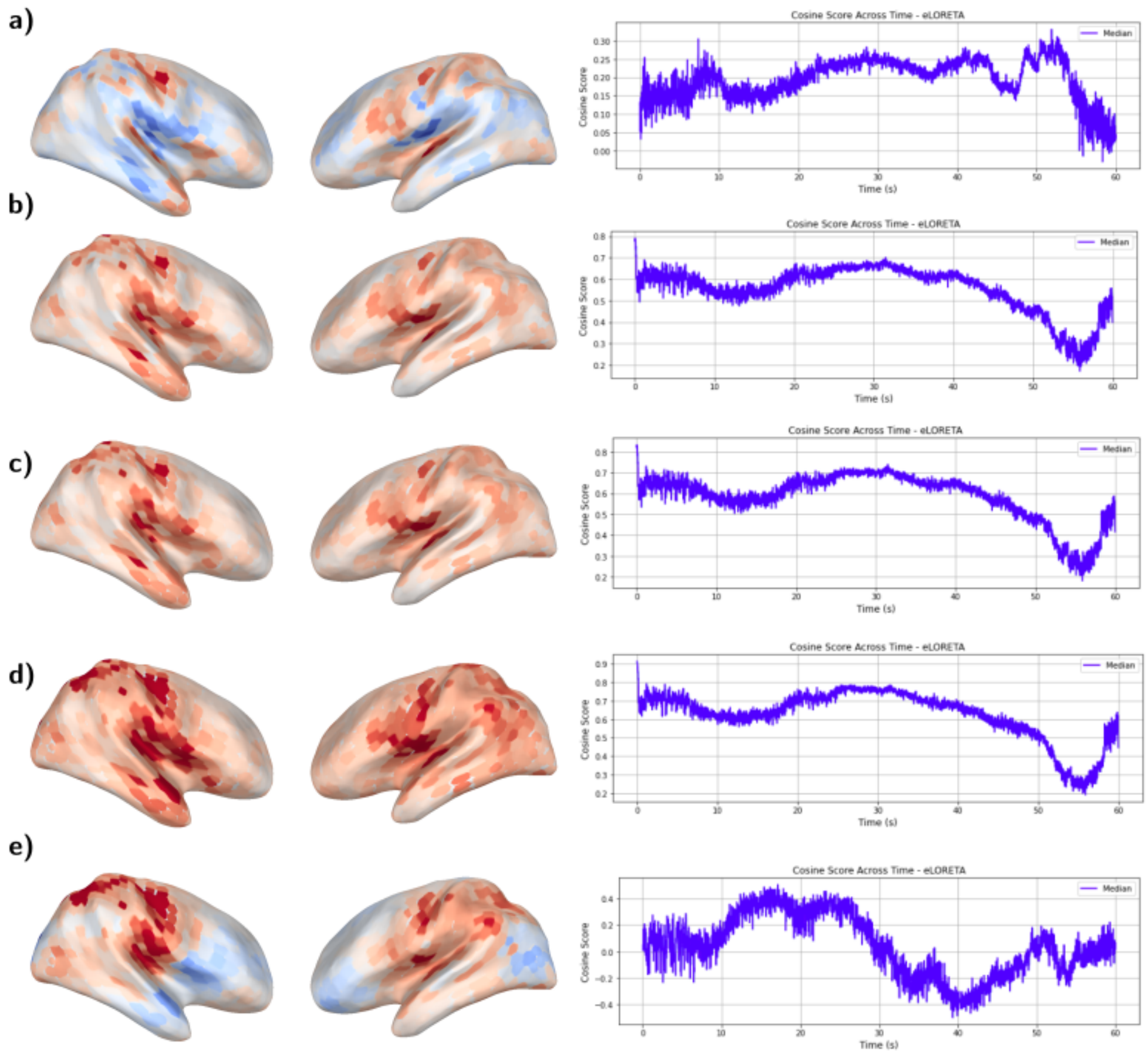

Figure S13: eLORETA method adjusting the loose parameter of a thalamic seizure with SNR 10. The localisation result at t=20s is visualised on the brain surface. The cosine score across the entire simulation is plotted beside. a) Loose = 0 (fixed orientation). b) Loose = 0, with np.abs(estimate). c) Loose = 0.05 (loose orientation). d) Loose = 1 (free orientation). e) Loose = 1 (free orientation), but taking only the normal to the surface component.

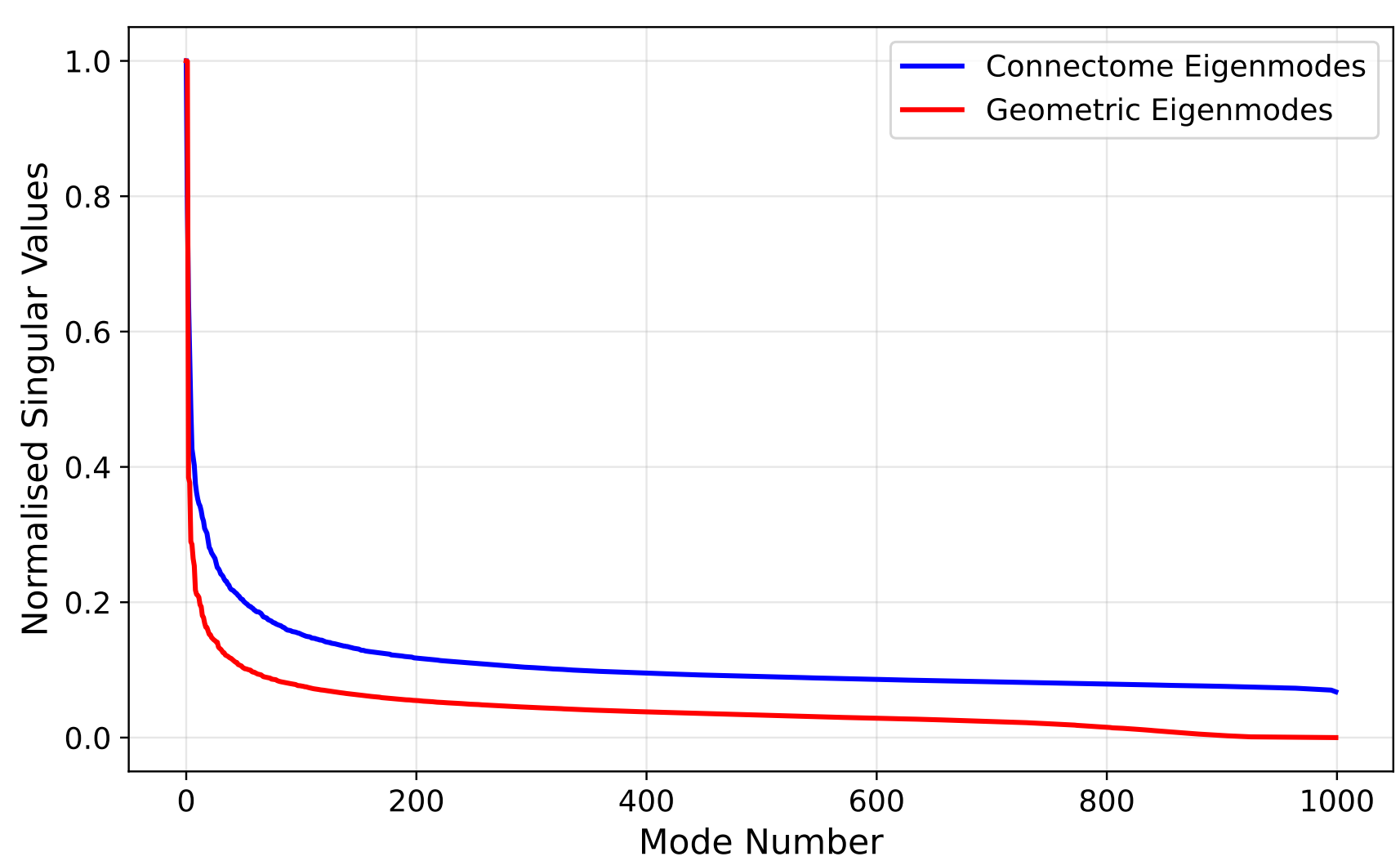

Figure S14: Singular value decomposition of eigenmode basis sets of 1000 modes in source space

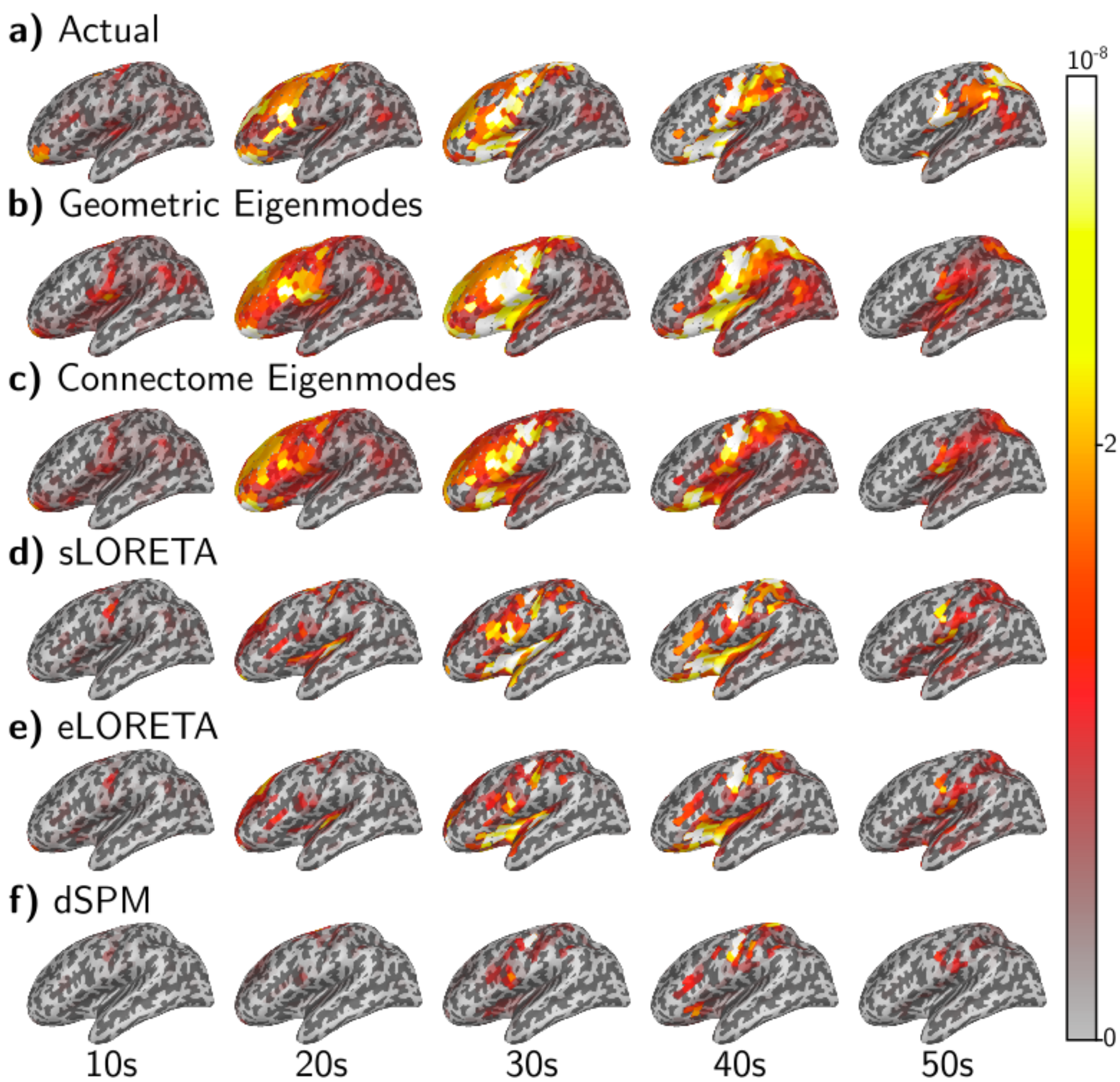

Figure S15: Seizure originating from cingulate cortex and partial spread ($g = 1.5$). All methods are kept at fixed orientation with no depth weighting.

source imaging framework. It is possible this may be due to the correlation between GEMs and CEMs, and in this supplementary section, we discuss the extent of the similarities between these two representations.

To illustrate this, we refer to Fig. 2c in Sina Mansour et al. [15], which compares the spatial similarity between geometric and connectome eigenmodes at high spatial resolution. The relevant panels are the middle and right subfigures, which differ in the connectome construction procedure. In both subfigures, after approximately the first 50 modes, the spatial similarity falls to around 25% for subsequent modes. While this still indicates a non-negligible correlation, it also demonstrates that GEM and CEM are sufficiently distinct to yield different performance characteristics, as observed in our results.

Furthermore, Fig. S11 from Sina Mansour et al. [15] assesses the Procrustes transform distance between the CEM and GEM subspaces. Their finding is that, even when considering linear combinations, the latent spaces explored by the connectome and geometry modes remain meaningfully distinct (differences of over 20%), while still exhibiting substantial overlap in the latent space they span (70-80%).

We note that their analysis was performed at a higher spatial resolution than the 1000-region parcellation used in our study. There is evidence that this divergence persists, and may even occur earlier, at our resolution. First, Fig. 3 in our paper shows clear differences in variance explained by GEM and CEM in both source and EEG space. Second, the singular value spectra from the SVD analysis of the eigenmodes in source space (Fig. S14) further indicate that the two bases distribute variance differently. Together, these results suggest that GEM and CEM are related, but not interchangeable representations at the resolution considered our study.

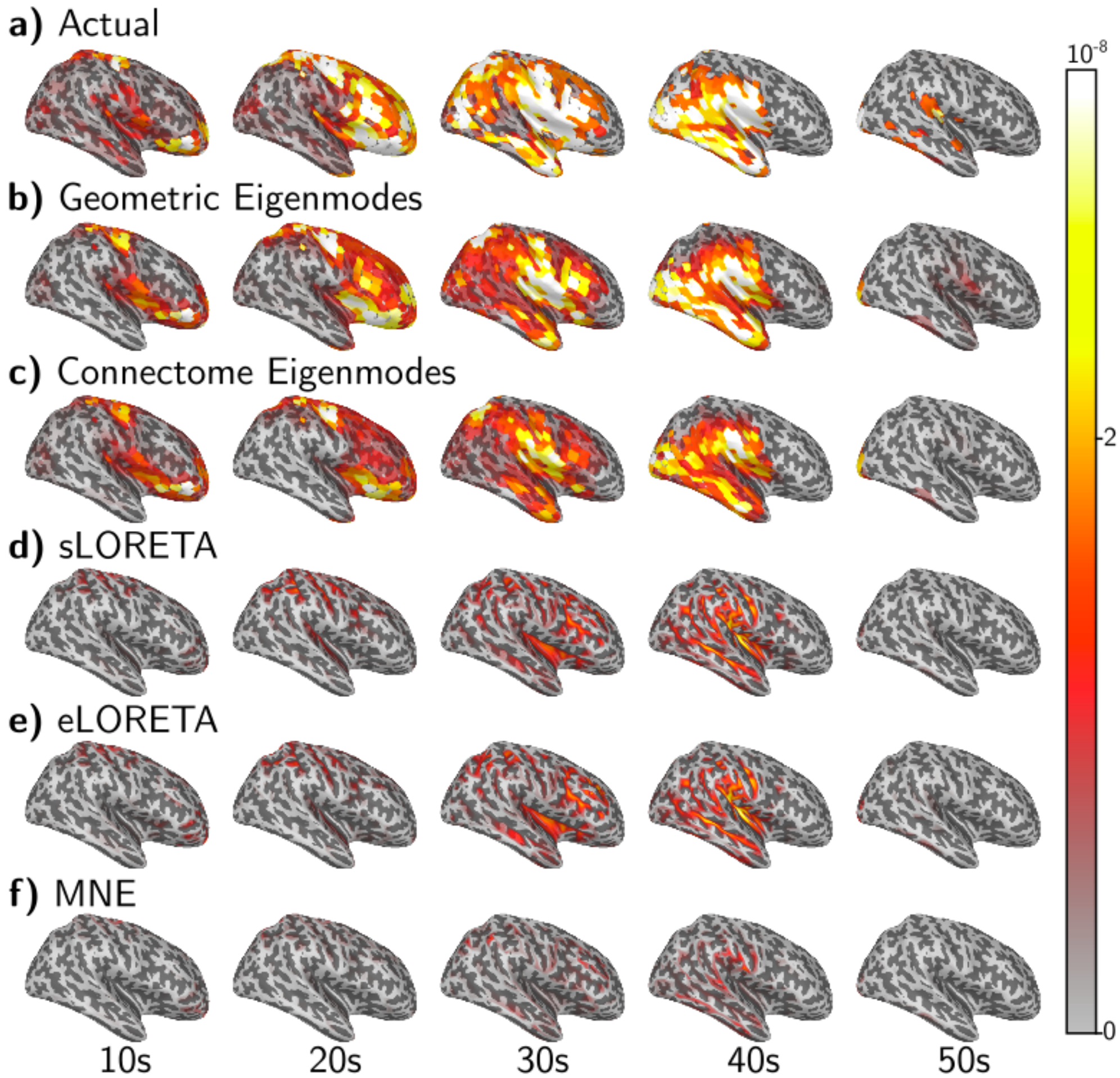

Figure S16: Source localisation example for seizure originating from thalamus plotted on right hemisphere 5 time points are selected in increments of 10s and visualised on the right hemisphere. 50% EEG power for this simulation occurs at the 30s. The colour scale is set such that maximum amplitude is white, progressing to yellow, orange, red, and transparent for 0. a) The actual or "ground truth" source activity as generated from the Epileptor model. We see that as time progresses, the seizure spreads from the anterior to posterior regions of the brain. During this time, different areas of the brain are activated in varying spatial extents and patterns. b) & c) Both geometric eigenmodes and connectome eigenmodes are able to perform reasonably well in sparse activations (10s and 50s), as well as dispersed activations (20s - 40s). d)-f) The LORETA family, predict more focal sources with default parameter settings and regularisation parameter set according to the $10^{-SNR/10}$ heuristic.

Table S1: Supporting Statistics for Results Fig.6

		RLE		RLE_point		cos		se		varexp_stc	
		mean	std	mean	std	mean	std	mean	std	mean	std
Analysis_type	SNR										
GEM_discrete	3.00	0.44	0.09	14.97	5.57	0.32	0.16	0.08	0.01	-0.31	0.50
	10.00	0.00	0.00	7.54	3.58	0.48	0.15	0.06	0.01	0.09	0.31
	None	0.12	0.14	4.05	2.08	0.76	0.07	0.03	0.00	0.58	0.10
GEM_discrete_subject_group	3.00	0.26	0.08	14.68	5.16	0.31	0.16	0.09	0.01	-0.45	0.60
	10.00	0.00	0.00	7.56	3.58	0.50	0.15	0.06	0.01	0.07	0.35
	None	0.12	0.15	4.05	2.15	0.76	0.07	0.03	0.00	0.58	0.10
GEM_discrete_group_group	3.00	1.12	0.29	11.69	3.22	0.22	0.15	0.09	0.01	-0.31	0.34
	10.00	0.00	0.01	10.04	5.99	0.47	0.13	0.07	0.01	-0.10	0.45
	None	0.07	0.08	4.26	1.74	0.55	0.05	0.05	0.01	0.27	0.04
SURROGATE_rotate_GEM_discrete	3.00	0.66	0.56	13.46	5.36	0.27	0.16	0.08	0.01	-0.32	0.46
	10.00	0.00	0.00	7.38	3.47	0.48	0.15	0.06	0.01	0.09	0.31
	None	0.11	0.14	4.04	2.08	0.75	0.07	0.03	0.00	0.58	0.10
SURROGATE_ROLL_GEM_discrete	3.00	0.61	0.13	5.87	1.45	0.13	0.04	0.07	0.02	-0.07	0.07
	10.00	0.36	0.10	5.01	1.40	0.19	0.05	0.07	0.02	0.01	0.04
	None	0.49	0.14	5.16	1.09	0.28	0.07	0.07	0.02	0.07	0.06
CEM_discrete	3.00	1.06	0.19	7.22	1.57	0.32	0.06	0.06	0.01	0.08	0.07
	10.00	0.20	0.08	5.39	1.65	0.46	0.07	0.06	0.01	0.18	0.08
	None	0.24	0.20	4.20	1.85	0.74	0.05	0.03	0.01	0.50	0.04
CEM_discrete_subject_group	3.00	0.96	0.12	8.36	1.91	0.36	0.06	0.07	0.01	0.02	0.15
	10.00	0.13	0.04	5.75	1.35	0.46	0.06	0.06	0.01	0.19	0.08
	None	0.29	0.27	4.22	1.96	0.75	0.05	0.03	0.01	0.53	0.05
CEM_discrete_group_group	3.00	1.74	0.19	6.60	1.25	0.29	0.04	0.07	0.01	0.01	0.08
	10.00	0.17	0.04	6.29	1.80	0.40	0.03	0.06	0.01	0.12	0.04
	None	0.16	0.11	4.41	1.28	0.44	0.03	0.06	0.01	0.18	0.02
SURROGATE_rotate_CEM_discrete	3.00	11.03	0.53	14.77	1.43	0.36	0.05	0.06	0.01	0.12	0.04
	10.00	4.80	1.58	10.07	2.51	0.46	0.03	0.06	0.02	0.20	0.03
	None	0.61	0.18	5.17	1.96	0.62	0.04	0.05	0.01	0.33	0.02
SURROGATE_ROLL_CEM_discrete	3.00	0.36	0.42	6.04	1.90	0.12	0.04	0.08	0.02	-0.13	0.09
	10.00	0.08	0.03	5.01	1.37	0.18	0.06	0.07	0.02	-0.01	0.05
	None	0.19	0.07	5.04	1.12	0.26	0.08	0.07	0.02	0.06	0.07
dSPM	3.00	0.00	0.00	6.88	1.54	0.13	0.01	0.08	0.02	-0.19	0.09
	10.00	0.00	0.00	6.18	2.08	0.16	0.01	0.08	0.02	-0.09	0.06
	None	2.62	0.58	6.50	1.16	0.23	0.02	0.07	0.02	0.05	0.01
MNE	3.00	0.08	0.02	5.17	1.27	0.17	0.01	0.07	0.02	-0.02	0.02
	10.00	0.25	0.06	4.95	1.28	0.20	0.02	0.07	0.02	0.01	0.01
	None	0.63	0.22	5.19	0.80	0.30	0.02	0.06	0.02	0.09	0.01
sLORETA	3.00	0.00	0.00	6.91	1.66	0.15	0.01	0.09	0.02	-0.30	0.12
	10.00	0.00	0.00	6.16	2.03	0.18	0.01	0.09	0.02	-0.24	0.09
	None	0.04	0.05	5.67	1.89	0.25	0.02	0.08	0.02	-0.10	0.05
eLORETA	3.00	0.00	0.00	6.75	1.62	0.14	0.01	0.09	0.02	-0.27	0.10
	10.00	0.00	0.00	5.98	1.88	0.18	0.02	0.08	0.02	-0.21	0.07
	None	0.01	0.01	5.33	1.57	0.27	0.02	0.07	0.02	-0.02	0.03

Table S2: Pairwise Wilcoxon Statistics of GEM against other Methods for Results Fig.6

	SNR	analysis_type	metric	wilcoxon_stat	wilcoxon_pval
0	None	GEM_discrete_subject_group	RLE	1175424.000000	1.000000
1	None	GEM_discrete_subject_group	RLE_point	1157984.000000	0.999996
2	None	GEM_discrete_subject_group	cos	669331.000000	1.000000
3	None	GEM_discrete_subject_group	se	843810.000000	0.000000
4	None	GEM_discrete_subject_group	varexp_stc	1243241.000000	0.000000

5	None	GEM_discrete_group_group	RLE	1436449.000000	1.000000
6	None	GEM_discrete_group_group	RLE_point	754133.000000	0.000000
7	None	GEM_discrete_group_group	cos	2077741.000000	0.000000
8	None	GEM_discrete_group_group	se	0.000000	0.000000
9	None	GEM_discrete_group_group	varexp_stc	2077741.000000	0.000000
10	None	CEM_discrete_subject_group	RLE	0.000000	0.000000
11	None	CEM_discrete_subject_group	RLE_point	548552.000000	0.000000
12	None	CEM_discrete_subject_group	cos	1600188.000000	0.000000
13	None	CEM_discrete_subject_group	se	280974.000000	0.000000
14	None	CEM_discrete_subject_group	varexp_stc	1790143.000000	0.000000
15	None	CEM_discrete	RLE	2683.000000	0.000000
16	None	CEM_discrete	RLE_point	656447.000000	0.000000
17	None	CEM_discrete	cos	2068112.000000	0.000000
18	None	CEM_discrete	se	678.000000	0.000000
19	None	CEM_discrete	varexp_stc	2077131.000000	0.000000
20	None	CEM_discrete_group_group	RLE	416150.000000	0.000000
21	None	CEM_discrete_group_group	RLE_point	691926.000000	0.000000
22	None	CEM_discrete_group_group	cos	2077741.000000	0.000000
23	None	CEM_discrete_group_group	se	0.000000	0.000000
24	None	CEM_discrete_group_group	varexp_stc	2077741.000000	0.000000
25	None	SURROGATE_ROLL_GEM_discrete	RLE	14.000000	0.000000
26	None	SURROGATE_ROLL_GEM_discrete	RLE_point	519190.000000	0.000000
27	None	SURROGATE_ROLL_GEM_discrete	cos	2077741.000000	0.000000
28	None	SURROGATE_ROLL_GEM_discrete	se	0.000000	0.000000
29	None	SURROGATE_ROLL_GEM_discrete	varexp_stc	2077741.000000	0.000000
30	None	dSPM	RLE	0.000000	0.000000
31	None	dSPM	RLE_point	12018.000000	0.000000
32	None	dSPM	cos	2077741.000000	0.000000
33	None	dSPM	se	0.000000	0.000000
34	None	dSPM	varexp_stc	2077741.000000	0.000000
35	None	MNE	RLE	0.000000	0.000000
36	None	MNE	RLE_point	354161.000000	0.000000
37	None	MNE	cos	2077741.000000	0.000000
38	None	MNE	se	0.000000	0.000000
39	None	MNE	varexp_stc	2077741.000000	0.000000
40	None	sLORETA	RLE	2002372.000000	1.000000
41	None	sLORETA	RLE_point	16150.000000	0.000000
42	None	sLORETA	cos	2077741.000000	0.000000
43	None	sLORETA	se	0.000000	0.000000
44	None	sLORETA	varexp_stc	2077741.000000	0.000000
45	None	eLORETA	RLE	2077737.000000	1.000000
46	None	eLORETA	RLE_point	80500.000000	0.000000
47	None	eLORETA	cos	2077741.000000	0.000000
48	None	eLORETA	se	0.000000	0.000000
49	None	eLORETA	varexp_stc	2077741.000000	0.000000
50	None	SURROGATE_ROLL_CEM_discrete	RLE	336891.500000	0.000000
51	None	SURROGATE_ROLL_CEM_discrete	RLE_point	521261.000000	0.000000
52	None	SURROGATE_ROLL_CEM_discrete	cos	2077741.000000	0.000000
53	None	SURROGATE_ROLL_CEM_discrete	se	0.000000	0.000000
54	None	SURROGATE_ROLL_CEM_discrete	varexp_stc	2077741.000000	0.000000
55	None	SURROGATE_rotate_GEM_discrete	RLE	2077741.000000	1.000000
56	None	SURROGATE_rotate_GEM_discrete	RLE_point	1060444.000000	0.791598
57	None	SURROGATE_rotate_GEM_discrete	cos	1197987.000000	0.000000
58	None	SURROGATE_rotate_GEM_discrete	se	929263.500000	0.000019
59	None	SURROGATE_rotate_GEM_discrete	varexp_stc	1142928.500000	0.000045
60	None	SURROGATE_rotate_CEM_discrete	RLE	7.000000	0.000000
61	None	SURROGATE_rotate_CEM_discrete	RLE_point	506971.000000	0.000000
62	None	SURROGATE_rotate_CEM_discrete	cos	2077741.000000	0.000000
63	None	SURROGATE_rotate_CEM_discrete	se	0.000000	0.000000

64	None	SURROGATE_rotate_CEM_discrete	varexp_stc	2077741.000000	0.000000
65	10.000000	GEM_discrete_subject_group	RLE	1666225.000000	1.000000
66	10.000000	GEM_discrete_subject_group	RLE_point	972916.000000	0.006525
67	10.000000	GEM_discrete_subject_group	cos	0.000000	1.000000
68	10.000000	GEM_discrete_subject_group	se	888683.000000	0.000000
69	10.000000	GEM_discrete_subject_group	varexp_stc	1276007.000000	0.000000
70	10.000000	GEM_discrete_group_group	RLE	1622435.000000	1.000000
71	10.000000	GEM_discrete_group_group	RLE_point	446568.000000	0.000000
72	10.000000	GEM_discrete_group_group	cos	1272154.000000	0.000000
73	10.000000	GEM_discrete_group_group	se	30911.000000	0.000000
74	10.000000	GEM_discrete_group_group	varexp_stc	2048096.000000	0.000000
75	10.000000	CEM_discrete_subject_group	RLE	0.000000	0.000000
76	10.000000	CEM_discrete_subject_group	RLE_point	1677913.000000	1.000000
77	10.000000	CEM_discrete_subject_group	cos	1548153.000000	0.000000
78	10.000000	CEM_discrete_subject_group	se	1456311.000000	1.000000
79	10.000000	CEM_discrete_subject_group	varexp_stc	553883.000000	1.000000
80	10.000000	CEM_discrete	RLE	0.000000	0.000000
81	10.000000	CEM_discrete	RLE_point	1785765.000000	1.000000
82	10.000000	CEM_discrete	cos	1531425.000000	0.000000
83	10.000000	CEM_discrete	se	1371470.000000	1.000000
84	10.000000	CEM_discrete	varexp_stc	560929.000000	1.000000
85	10.000000	CEM_discrete_group_group	RLE	0.000000	0.000000
86	10.000000	CEM_discrete_group_group	RLE_point	1515740.000000	1.000000
87	10.000000	CEM_discrete_group_group	cos	1593187.000000	0.000000
88	10.000000	CEM_discrete_group_group	se	597087.000000	0.000000
89	10.000000	CEM_discrete_group_group	varexp_stc	797963.000000	1.000000
90	10.000000	SURROGATE_ROLL_GEM_discrete	RLE	0.000000	0.000000
91	10.000000	SURROGATE_ROLL_GEM_discrete	RLE_point	1780838.000000	1.000000
92	10.000000	SURROGATE_ROLL_GEM_discrete	cos	2077741.000000	0.000000
93	10.000000	SURROGATE_ROLL_GEM_discrete	se	18207.000000	0.000000
94	10.000000	SURROGATE_ROLL_GEM_discrete	varexp_stc	1162487.000000	0.000000
95	10.000000	dSPM	RLE	1666225.000000	1.000000
96	10.000000	dSPM	RLE_point	1574422.000000	1.000000
97	10.000000	dSPM	cos	2077741.000000	0.000000
98	10.000000	dSPM	se	426709.000000	0.000000
99	10.000000	dSPM	varexp_stc	1643614.000000	0.000000
100	10.000000	MNE	RLE	0.000000	0.000000
101	10.000000	MNE	RLE_point	1846305.000000	1.000000
102	10.000000	MNE	cos	2077741.000000	0.000000
103	10.000000	MNE	se	515837.000000	0.000000
104	10.000000	MNE	varexp_stc	1513911.000000	0.000000
105	10.000000	sLORETA	RLE	1666225.000000	1.000000
106	10.000000	sLORETA	RLE_point	1583493.000000	1.000000
107	10.000000	sLORETA	cos	2077741.000000	0.000000
108	10.000000	sLORETA	se	25539.000000	0.000000
109	10.000000	sLORETA	varexp_stc	2055372.000000	0.000000
110	10.000000	eLORETA	RLE	1666225.000000	1.000000
111	10.000000	eLORETA	RLE_point	1642845.000000	1.000000
112	10.000000	eLORETA	cos	2077741.000000	0.000000
113	10.000000	eLORETA	se	100142.000000	0.000000
114	10.000000	eLORETA	varexp_stc	1977776.000000	0.000000
115	10.000000	SURROGATE_ROLL_CEM_discrete	RLE	0.000000	0.000000
116	10.000000	SURROGATE_ROLL_CEM_discrete	RLE_point	1751661.500000	1.000000
117	10.000000	SURROGATE_ROLL_CEM_discrete	cos	2077741.000000	0.000000
118	10.000000	SURROGATE_ROLL_CEM_discrete	se	13990.500000	0.000000
119	10.000000	SURROGATE_ROLL_CEM_discrete	varexp_stc	1088598.500000	0.018677
120	10.000000	SURROGATE_rotate_GEM_discrete	RLE	2054950.000000	1.000000
121	10.000000	SURROGATE_rotate_GEM_discrete	RLE_point	1108466.000000	0.995596
122	10.000000	SURROGATE_rotate_GEM_discrete	cos	1109279.500000	0.004024

123	10.000000	SURROGATE_rotate_GEM_discrete	se	910050.500000	0.000001
124	10.000000	SURROGATE_rotate_GEM_discrete	varexp_stc	1127116.000000	0.000448
125	10.000000	SURROGATE_rotate_CEM_discrete	RLE	0.000000	0.000000
126	10.000000	SURROGATE_rotate_CEM_discrete	RLE_point	264385.000000	0.000000
127	10.000000	SURROGATE_rotate_CEM_discrete	cos	962463.000000	0.998282
128	10.000000	SURROGATE_rotate_CEM_discrete	se	262730.000000	0.000000
129	10.000000	SURROGATE_rotate_CEM_discrete	varexp_stc	538598.000000	1.000000
130	3.000000	GEM_discrete_subject_group	RLE	2070328.000000	1.000000
131	3.000000	GEM_discrete_subject_group	RLE_point	1121544.000000	0.999070
132	3.000000	GEM_discrete_subject_group	cos	1437593.000000	0.000000
133	3.000000	GEM_discrete_subject_group	se	20109.000000	0.000000
134	3.000000	GEM_discrete_subject_group	varexp_stc	2054607.000000	0.000000
135	3.000000	GEM_discrete_group_group	RLE	13467.000000	0.000000
136	3.000000	GEM_discrete_group_group	RLE_point	1837161.000000	1.000000
137	3.000000	GEM_discrete_group_group	cos	2077741.000000	0.000000
138	3.000000	GEM_discrete_group_group	se	709630.000000	0.000000
139	3.000000	GEM_discrete_group_group	varexp_stc	1026057.000000	0.685194
140	3.000000	CEM_discrete_subject_group	RLE	4.000000	0.000000
141	3.000000	CEM_discrete_subject_group	RLE_point	2031249.000000	1.000000
142	3.000000	CEM_discrete_subject_group	cos	621541.000000	1.000000
143	3.000000	CEM_discrete_subject_group	se	1762105.000000	1.000000
144	3.000000	CEM_discrete_subject_group	varexp_stc	315255.000000	1.000000
145	3.000000	CEM_discrete	RLE	56.000000	0.000000
146	3.000000	CEM_discrete	RLE_point	2049882.000000	1.000000
147	3.000000	CEM_discrete	cos	815946.000000	1.000000
148	3.000000	CEM_discrete	se	1667576.000000	1.000000
149	3.000000	CEM_discrete	varexp_stc	409549.000000	1.000000
150	3.000000	CEM_discrete_group_group	RLE	22.000000	0.000000
151	3.000000	CEM_discrete_group_group	RLE_point	2057487.000000	1.000000
152	3.000000	CEM_discrete_group_group	cos	1184472.000000	0.000000
153	3.000000	CEM_discrete_group_group	se	1507125.000000	1.000000
154	3.000000	CEM_discrete_group_group	varexp_stc	561369.000000	1.000000
155	3.000000	SURROGATE_ROLL_GEM_discrete	RLE	19507.000000	0.000000
156	3.000000	SURROGATE_ROLL_GEM_discrete	RLE_point	2026583.500000	1.000000
157	3.000000	SURROGATE_ROLL_GEM_discrete	cos	1990109.000000	0.000000
158	3.000000	SURROGATE_ROLL_GEM_discrete	se	1616816.000000	1.000000
159	3.000000	SURROGATE_ROLL_GEM_discrete	varexp_stc	765898.000000	1.000000
160	3.000000	dSPM	RLE	2077741.000000	1.000000
161	3.000000	dSPM	RLE_point	2052984.000000	1.000000
162	3.000000	dSPM	cos	1892610.000000	0.000000
163	3.000000	dSPM	se	1241900.000000	1.000000
164	3.000000	dSPM	varexp_stc	656579.000000	1.000000
165	3.000000	MNE	RLE	2077741.000000	1.000000
166	3.000000	MNE	RLE_point	2060664.000000	1.000000
167	3.000000	MNE	cos	1687206.000000	0.000000
168	3.000000	MNE	se	1506108.000000	1.000000
169	3.000000	MNE	varexp_stc	548761.000000	1.000000
170	3.000000	sLORETA	RLE	2077741.000000	1.000000
171	3.000000	sLORETA	RLE_point	2054839.000000	1.000000
172	3.000000	sLORETA	cos	1819943.000000	0.000000
173	3.000000	sLORETA	se	561722.000000	0.000000
174	3.000000	sLORETA	varexp_stc	865965.000000	1.000000
175	3.000000	eLORETA	RLE	2077741.000000	1.000000
176	3.000000	eLORETA	RLE_point	2055798.000000	1.000000
177	3.000000	eLORETA	cos	1823989.000000	0.000000
178	3.000000	eLORETA	se	617305.000000	0.000000
179	3.000000	eLORETA	varexp_stc	790207.000000	1.000000
180	3.000000	SURROGATE_ROLL_CEM_discrete	RLE	2072653.000000	1.000000
181	3.000000	SURROGATE_ROLL_CEM_discrete	RLE_point	1879859.000000	1.000000

182	3.000000	SURROGATE_ROLL_CEM_discrete	cos	2073377.000000	0.000000
183	3.000000	SURROGATE_ROLL_CEM_discrete	se	1262177.000000	1.000000
184	3.000000	SURROGATE_ROLL_CEM_discrete	varexp_stc	993275.000000	0.968209
185	3.000000	SURROGATE_rotate_GEM_discrete	RLE	306851.000000	0.000000
186	3.000000	SURROGATE_rotate_GEM_discrete	RLE_point	1390609.500000	1.000000
187	3.000000	SURROGATE_rotate_GEM_discrete	cos	1529999.500000	0.000000
188	3.000000	SURROGATE_rotate_GEM_discrete	se	765643.000000	0.000000
189	3.000000	SURROGATE_rotate_GEM_discrete	varexp_stc	1249813.500000	0.000000
190	3.000000	SURROGATE_rotate_CEM_discrete	RLE	0.000000	0.000000
191	3.000000	SURROGATE_rotate_CEM_discrete	RLE_point	1143392.500000	0.999959
192	3.000000	SURROGATE_rotate_CEM_discrete	cos	628124.000000	1.000000
193	3.000000	SURROGATE_rotate_CEM_discrete	se	2058000.000000	1.000000
194	3.000000	SURROGATE_rotate_CEM_discrete	varexp_stc	501320.000000	1.000000
